# Is the traction table necessary to treat femoral fractures with intramedullary nailing? A meta-analysis

**DOI:** 10.1186/s13018-023-03659-y

**Published:** 2023-04-05

**Authors:** Yu-kun He, Yi-chong Wang, Feng-feng Li

**Affiliations:** 1 Department of Orthopedic Surgery, The Second Affiliated Hospital of Guangzhou Medical University, Guangzhou, 510000 Guangdong China; 2 Department of Human Structure and Repair, Faculty of Medicine and Health Sciences, Ghent University, 9000 Ghent, Belgium; 3 Department of Orthopedic Surgery, Nanjing Drum Tower Hospital, The Affiliated Hospital of Nanjing University Medical School, 210008, Nanjing, China

**Keywords:** Femoral fracture, Intramedullary nail, Traction table

## Abstract

**Background:**

The traction table is generally used in femoral intramedullary nailing surgery. Recently, some published studies have shown that the same or better treatment effects can be gotten without a traction table. It remains no consensus on this issue.

**Methods:**

The Preferred Reporting Items for Systematic Reviews and Meta-analyses guideline was applied in this study. We searched PubMed, Embase, Web of Science, and Cochrane Library databases for eligible studies. The random-effect model was used to calculate the standardized mean difference (SMD) and risk ratios with 95% CIs. Trial sequential analysis (TSA) was performed to verify the results.

**Results:**

The pooled estimates of seven studies, including 266 cases each in the manual traction group and traction table group, indicated that manual traction could shorten operative time [SMD, − 0.77; 95% CI (− 0.98, − 0.55); *P* < 0.00001] and preoperative set-up time [SMD, − 2.37; 95% CI (− 3.90, − 0.84); *P* = 0.002], but it would not reduce intraoperative blood loss volume and fluoroscopy time. No statistical difference was found in their fracture healing time, postoperative Harris scores, and malunion rate. The use of a Traction repositor could reduce the set-up time [SMD, − 2.48; 95% CI (− 4.91, − 0.05); *P* < 0.00001].

**Conclusions:**

Compared with manual traction, the traction table in femoral intramedullary nailing surgery lengthened operative time and preoperative set-up time. At the same time, it did not show significant advantages in reducing blood loss volume and fluoroscopy time, or improving prognosis. In clinical practice, the optimal surgical plan must be made on a case-by-case basis to avoid unnecessary traction table use.

**Supplementary Information:**

The online version contains supplementary material available at 10.1186/s13018-023-03659-y.

## Background

Femoral fracture is one of the most common fractures. Due to the traction of soft tissue around the femur, most patients have an obvious displacement of fractures that require surgery [1]. However, improper treatment often results in various complications, affecting patients’ quality of life [2]. Professor Kuntscher first used intramedullary nails to treat femoral shaft fractures in 1939. Since that, intramedullary nailing has gradually become the preferred method for femoral fracture due to its many advantages like simplified operation, causing less damage and early weight-bearing [3].

Closed reduction is the key and the difficulty to successfully placing intramedullary nails [4]. The traction table has been widely used in the reduction of lower limb fractures because of its obvious advantages: higher traction force, easier fluoroscopy and better stability, and the ability to maintain the force line. Nevertheless, its shortcomings are also noticeable. First, the position of the hip joint is forced to be neutral or abducted after reduction. Inserting the intramedullary nail is difficult in that position, especially through the trochanteric fossa approach. Second, the traction table can only provide axial traction, which cannot reduce various angular displacements. In femoral fractures, the gluteus medius and gluteus minimus will abduct the proximal fracture block, and the adductor muscle will pull the distal fracture. The iliopsoas muscle will flex and externally rotate the fracture fragments. Gravity also affects the anterior femoral arch angle [5, 6]. Third, the inappropriate use of the traction table may cause various complications like perineal injury [7]. Recently, studies showed that the same or better prognosis could be acquired without a traction table. A survey of orthopedic surgeons showed that there is still no consensus on this issue [8]. That is why we conducted this meta-analysis.

## Methods

### Study search and selection

We searched the PubMed, Embase, Web of Science, and Cochrane Library databases for related articles published until August 30, 2022. The search strategy was as follows: (femor* OR femur* intertrochanter* OR subtrochanter*) AND fractur* AND ([tract* AND (bed OR table OR frame)] OR lateral) AND nail* (refer to Additional file 1: Appendix Table 1). There were two reviewers independently assessing the papers. A third reviewer would make the final decision if they could not achieve the agreement through discussion. The inclusion criteria were as follows:

1. Randomized controlled trials (RCTs);
2. The study object was femoral fracture patients treated with the intramedullary nail and was 18 years of age or older;
3. The study compared the difference in operative procedures or prognosis between using the traction table and manual traction;
4. Sufficient data presented to allow further analysis;
5. Data not duplicated in another manuscript (refer to Table 1).

**Table 1:** Inclusion and exclusion criteria of the current meta-analysis

Detailed inclusion and exclusion criteria based on PICOS framework	
Populations	Femoral fracture patients treated with intramedullary nail and was 18 years of age or older
Intervention/Exposure	Using regular table during operation
Control	Using traction table during operation
Clinical outcomes	Operative time, blood loss volume, set-up time, fluoroscopy time, fracture healing time, Harris score, malunion rate
Study design	Randomized controlled trials (RCTs)
Exclusion criteria	Reviews
	Not RCTs
	Conference abstracts

Besides, we found some cohort studies compared the differences between using traction repositor and traction tables during screening. We also selected them for further analysis, referring to the above criteria.

### Data extraction and quality assessment

We used Microsoft Excel (Microsoft Corporation, USA) to compile the needed data. The GRADE (Grade of Recommendations Assessment, Development and Evaluation) guidelines were used to rate the quality of evidence, and we assessed RCTs' bias risk by the Cochrane Collaboration tool. The Newcastle–Ottawa Scale (NOS) score assessed the quality of cohort studies. Two evaluators conducted the independent evaluation.

### Statistical analysis

We divided each RCT patient into two groups: “Manual traction” and “Traction table.” To improve the accuracy of the results, we further divided “Manual traction” into two subgroups: “Lateral position” and “Supine position” because we found there are two types of manual traction surgery in these studies. The Std. Mean difference (SMD) or risk ratios (RR) assessed their effects. We pooled continuous data by inverse variance and used the Mantel–Haenszel method for dichotomous data. The random-effect model for anticipated heterogeneity determined all outcomes. The statistics *I*^2^ > 50% indicated the high heterogeneity, and *P* < 0.05 indicated the statistical differences of included studies. The sensitivity analyses would be performed for the results with high heterogeneity by using different statistical methods or excluding the source of heterogeneity. Trial sequential analysis (TSA) was performed to verify the positive results. The analysis of the “Traction repository” group and “Traction table” group of cohort studies was also carried out as described above by Review Manager 5.4.

## Results

### Study selection

In total, 2764 studies were screened from the four databases. Forty-eight relevant studies for further assessment. We excluded eight reviews, twenty-seven studies not including relevant data and six studies not RCTs. No other useful studies could be found from the references or other sources. At last, seven RCTs were included in the meta-analysis (refer to Additional file 3: Appendix Figure 1) [9–15]. Besides, we screened four cohort studies related to traction repositor by the same method [16–19].

### Study characteristics

**Fig. 1:**
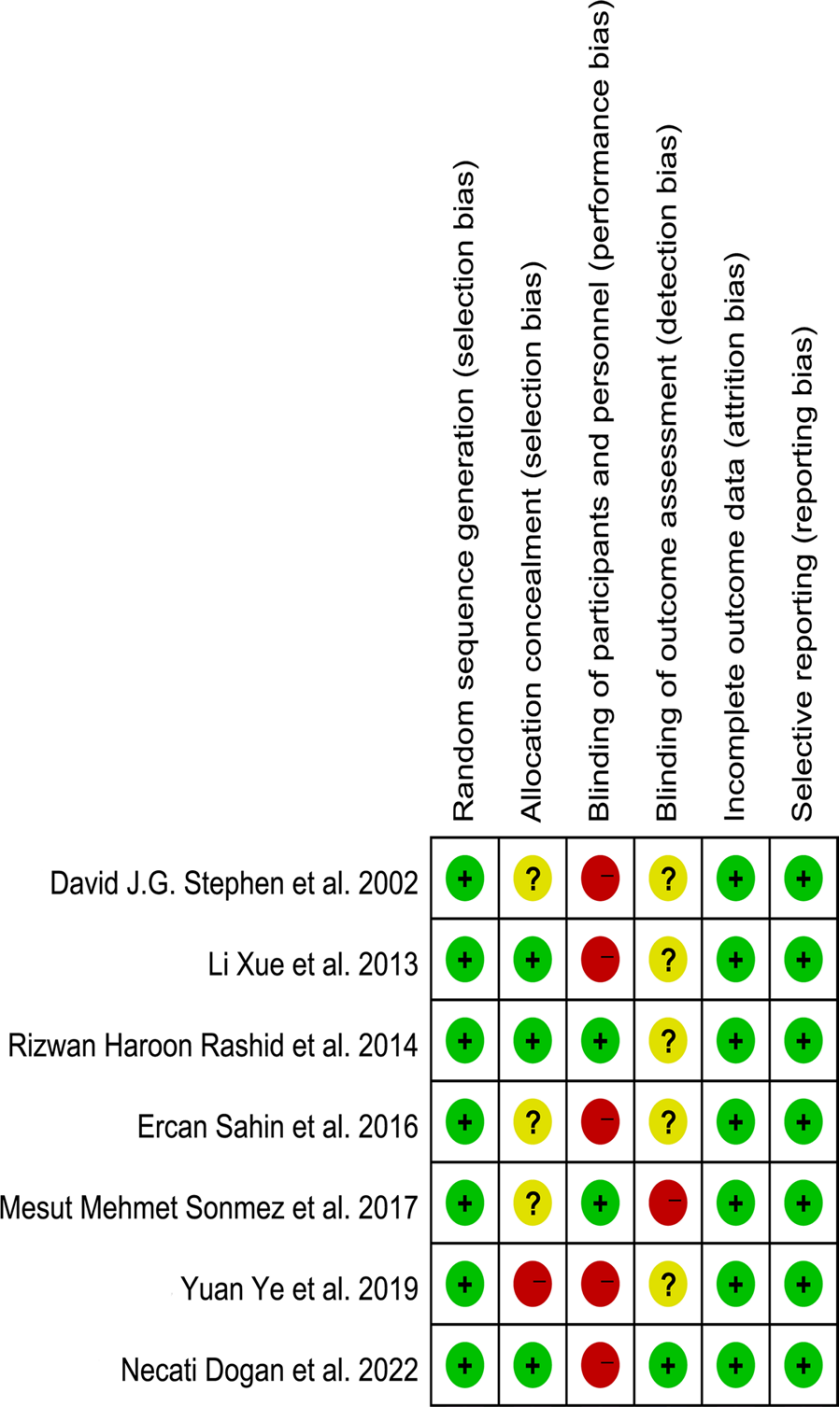
Summary of bias risk

The sample size of included studies ranged from 17 to 74. Intramedullary nails are used for all patients. The baseline characteristics of RCTs and cohort studies are shown in Table 2 and 3. In Fig. 1, the risk of bias was summarized. The GRADE ratings of RCTs are moderate because of the lack of blinding and the small sample size. The qualities of cohort studies are high according to their NOS scores (refer to Additional file 2: Appendix Table 2). The funnel plot was not feasible because of the few included studies.

**Table 2:** The baseline characteristics of RCTs

Study characteristics				Patients characteristics (Expose/control)				
Author/year	Study location	Period of enrollment	Study design	Subjects	Age	Male	Fracture type	Right side
Stephen et al. [9]	Canada	1997–2000	RCT	45/42	30 ± 15/34 ± 14	26/31	Femoral shaft fractures	23/23
Xue et al. [10]	China	2009–2010	RCT	60/60	77.3/75.7	26/29	Intertrochanteric fractures	21/23
Rashid et al. [11]	Karachi	2012–2013	RCT	37/37	36 ± 16.17/ 38 ± 17.73	26/29	Femoral shaft fractures	21/20
Sahin et al. [12]	Turkey	2014–2014	RCT	30/34	76.5 ± 10.2/74.8 ± 10.5	11/18	Intertrochanteric fractures	17/21
Sonmez et al. [13]	Turkey	2011–2013	RCT	37/36	78 ± 6/78 ± 6	-	Intertrochanteric fractures	–
Yuan et al. [14]	China	2015–2018	RCT	17/17	43.00 ± 14.73/44.12 ± 12.77	10/9	Femoral shaft fractures	–
Dogan et al. [15]	Turkey	2018–2019	RCT	40/40	81.45 ± 8.21/79.95 ± 8.23	12/11	Intertrochanteric fractures	18/18

**Table 3:** The baseline characteristics of cohort studies

Study characteristics				Patients characteristics (Expose/control)				
Author/year	Study location	Period of enrollment	Study design	Subjects	Age	Male	Fracture type	Right side
Zhang et al. [16]	China	2012–2015	RCT	48/74	39.85 ± 9.88/41.05 ± 11.47	29/43	Femoral shaft fractures	–
Du et al. [17]	China	2016–2018	RCT	44/42	70.8(60–86)/72.2(60–88)	12/11	Intertrochanteric fractures	23/20
Zhao et al. [18]	China	2017–2017	RCT	30/36	79.5 ± 9.0/79.2 ± 9.0	6/15	Intertrochanteric fractures	25/41
Yan et al. [19]	China	2015–2018	RCT	56/39	74.2 ± 12.2/78.8 ± 10.3	34/30	Intertrochanteric fractures	–

### Perioperative outcomes

#### operative time

**Fig. 2:**
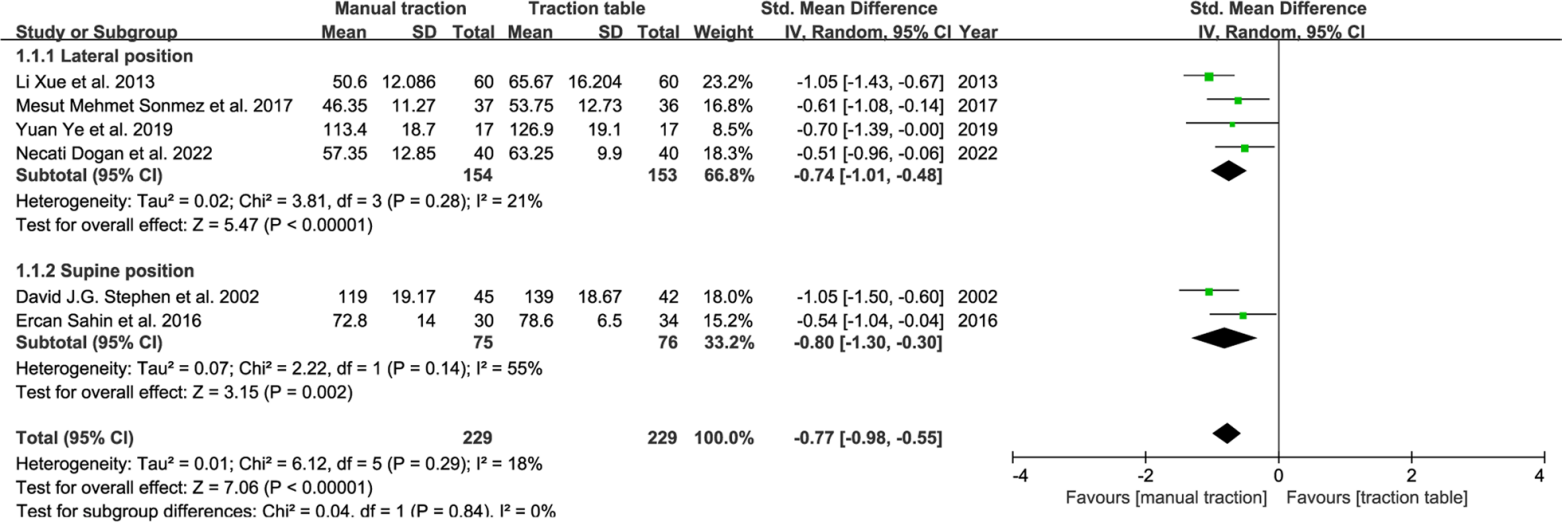
Forest plot summary comparing the operative time between the manual traction and traction table groups. CI = confidence interval, SMD = Std. Mean difference

**Fig. 3:**
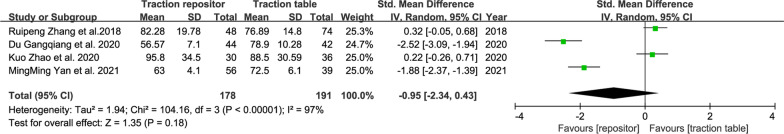
Forest plot summary comparing the operative time between the traction repositor and traction table groups

Six RCTs reported the average operative time. We divided them into two subgroups according to the operative position. The random-effect model was used for analysis. The results: SMD, − 0.77; 95% CI (− 0.98, − 0.55); *P* < 0.00001 (Fig. 2). The average operative time of the manual traction group was less than that of the traction table group. Also, four cohort studies compared the difference in operative time between the use of traction repositor and traction table in the supine position. We also analyzed them in the same way, and the results showed no statistical difference between the traction repositor group and the traction table group (Fig. 3).

#### Blood loss volume

**Fig. 4:**
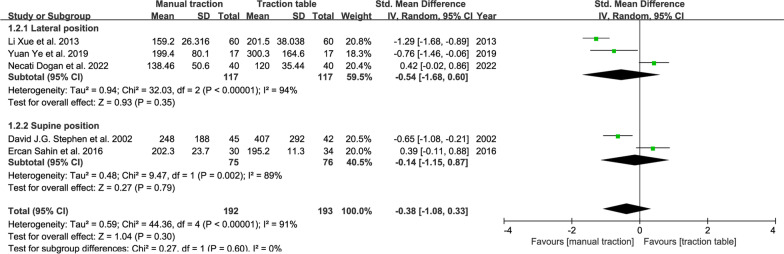
Forest plot summary comparing the blood loss volume between the manual traction and traction table groups

**Fig. 5:**
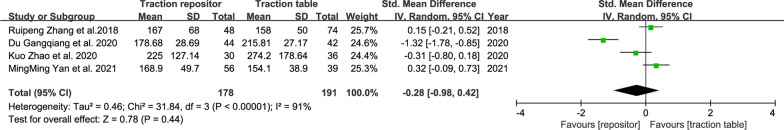
Forest plot summary comparing the blood loss volume between the traction repositor and traction table groups

Five RCTs reported intraoperative blood loss volume. We also divided them into two subgroups to analyze the random-effect model. The results: SMD, − 0.38; 95% CI (− 1.08, 0.33); *P* = 0.30 > 0.05 (Fig. 4). The average blood loss volume of the manual traction group did not have a statistical difference from that of the traction table group. Also, the analysis of cohort studies showed no statistical difference between the traction repositor group and the traction table group in blood loss volume (Fig. 5).

#### Set-up time

**Fig. 6:**
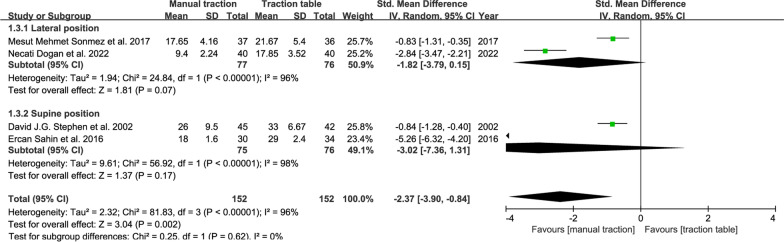
Forest plot summary comparing the set-up time between the manual traction and traction table groups

**Fig. 7:**
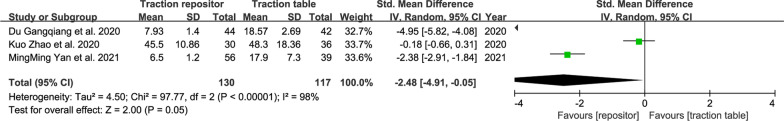
Forest plot summary comparing the set-up time between the traction repositor and traction table groups

Set-up time, defined as the time from anesthesia to wound incision, represents fracture reduction time. The results: Manual traction: 4 RCTs: SMD, − 2.37; 95% CI (− 3.90, − 0.84); *P* = 0.002 < 0.05 (Fig. 6). Traction repositor: three studies; SMD, − 2.48; 95% CI (− 4.91, − 0.05); *P* = 0.05 (Fig. 7). The average reduction time of manual traction was shorter than that of the traction table group. However, there is no statistical difference between the traction repositor group and the traction table group.

#### Fluoroscopy time

**Fig. 8:**
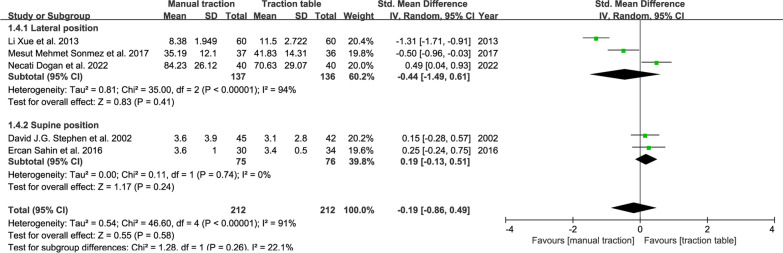
Forest plot summary comparing the fluoroscopy time between the manual traction and traction table groups

Five RCTs reported related indicators of intraoperative fluoroscopy time. The random-effect model results indicated no difference statistically: SMD, − 0.19; 95% CI (− 0.86, 0.49); *P* = 0.58 (Fig. 8).

### Prognostic outcomes

#### Fracture healing time

**Fig. 9:**
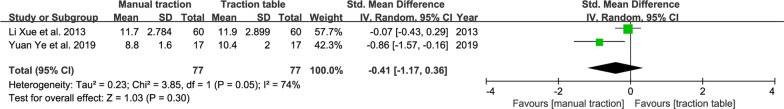
Forest plot summary comparing the fracture healing time between the manual traction and traction table groups

**Fig. 10:**
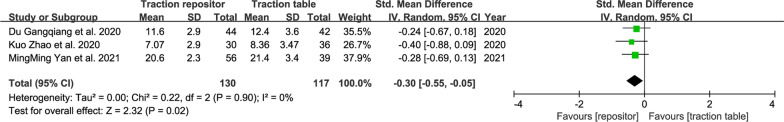
Forest plot summary comparing the fracture healing time between the traction repositor and traction table groups

The results: Manual traction: 4 RCTs: SMD, − 0.41; 95% CI (− 1.17, 0.36); *P* = 0.30 (Fig. 9). Traction repositor: three studies; SMD, − 0.30; 95% CI (− 0.55, − 0.05); *P* = 0.02 < 0.05 (Fig. 10). The fracture healing time of the traction repositor group was shorter than that of the traction table group.

#### Harris score

**Fig. 11:**
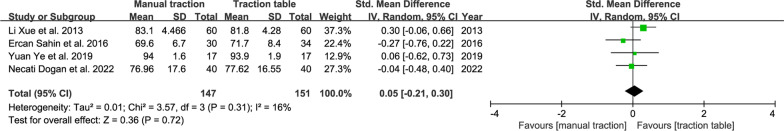
Forest plot summary comparing the Harris score between the manual traction and traction table groups

**Fig. 12:**
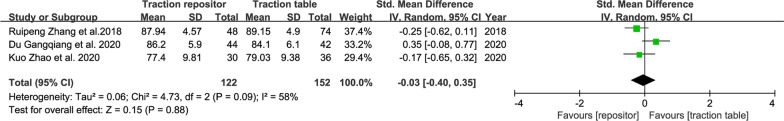
Forest plot summary comparing the Harris score between the traction repositor and traction table groups

Seven studies reported postoperative Harris scores, and the analysis results: Manual traction: four studies; SMD, 0.05; 95% CI (− 0.21, 0.30); *P* = 0.72 > 0.05 (Fig. 11); Traction repositor: three studies; SMD, − 0.03; 95% CI (− 0.40, 0.35); *P* = 0.88 > 0.05 (Fig. 12). Neither manual traction nor traction repositor could affect patients' joint function prognosis.

### Quality of fracture reduction

**Fig. 13:**
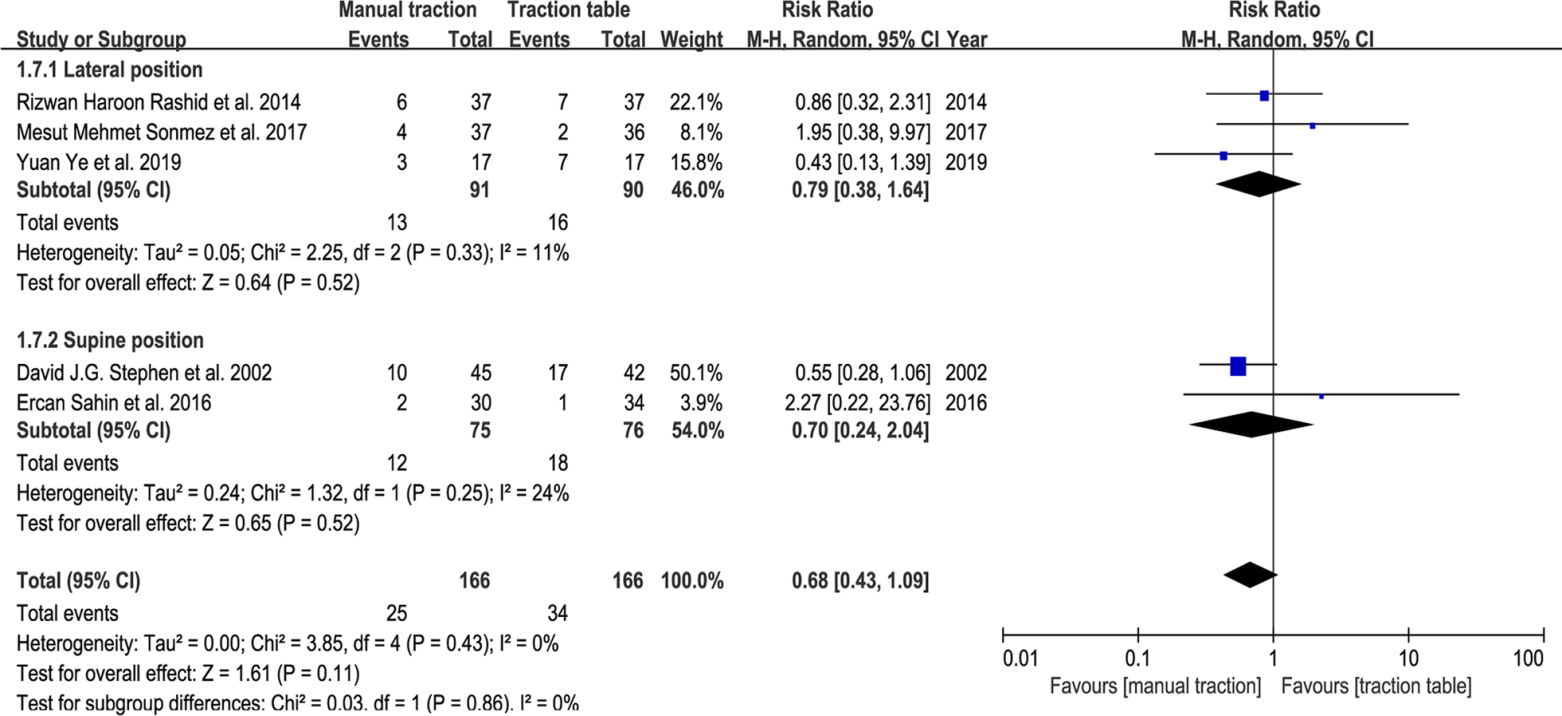
Forest plot summary comparing the malunion rate between the manual traction and traction table groups. RR = risk ratio

**Fig. 14:**
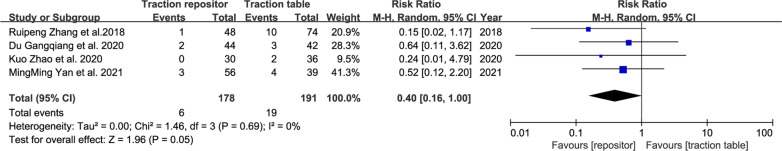
Forest plot summary comparing the malunion rate between the traction repositor and traction table groups

Seven articles counted the cases of malunion patients, including obvious angular displacement and shortening displacement. The results (Manual traction: 5 studies; RR, 0.68; 95% CI (0.43, 1.09); *P* = 0.11 > 0.05, Fig. 13; Traction repositor: 4 studies; RR, 0.40; 95% CI (0.16, 1.00); *P* = 0.05, Fig. 14). It showed that using the traction table did not affect the malunion rate.

### Trial sequential analysis

**Fig. 15:**
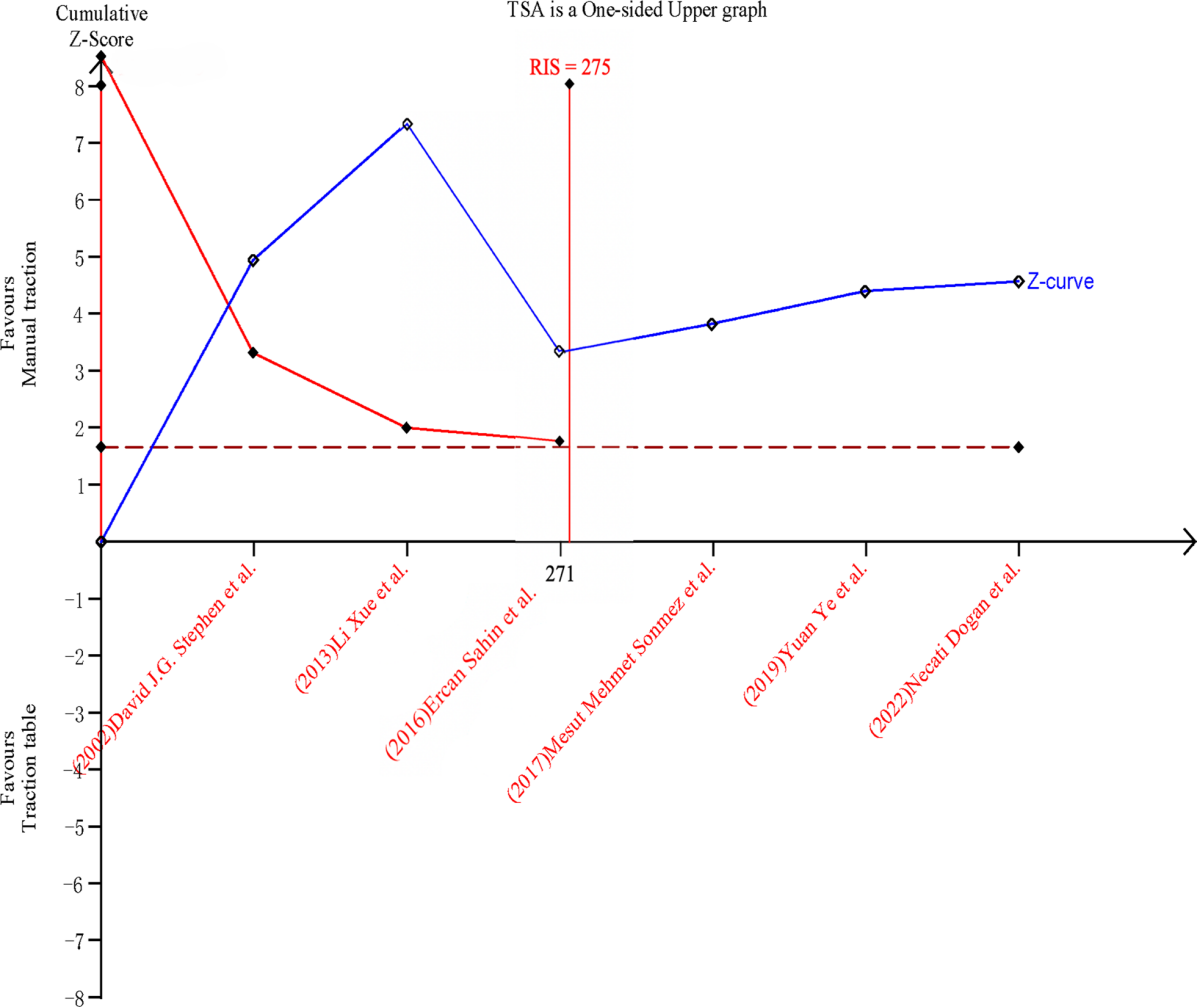
Sequential analysis of the effect on operative time

**Fig. 16:**
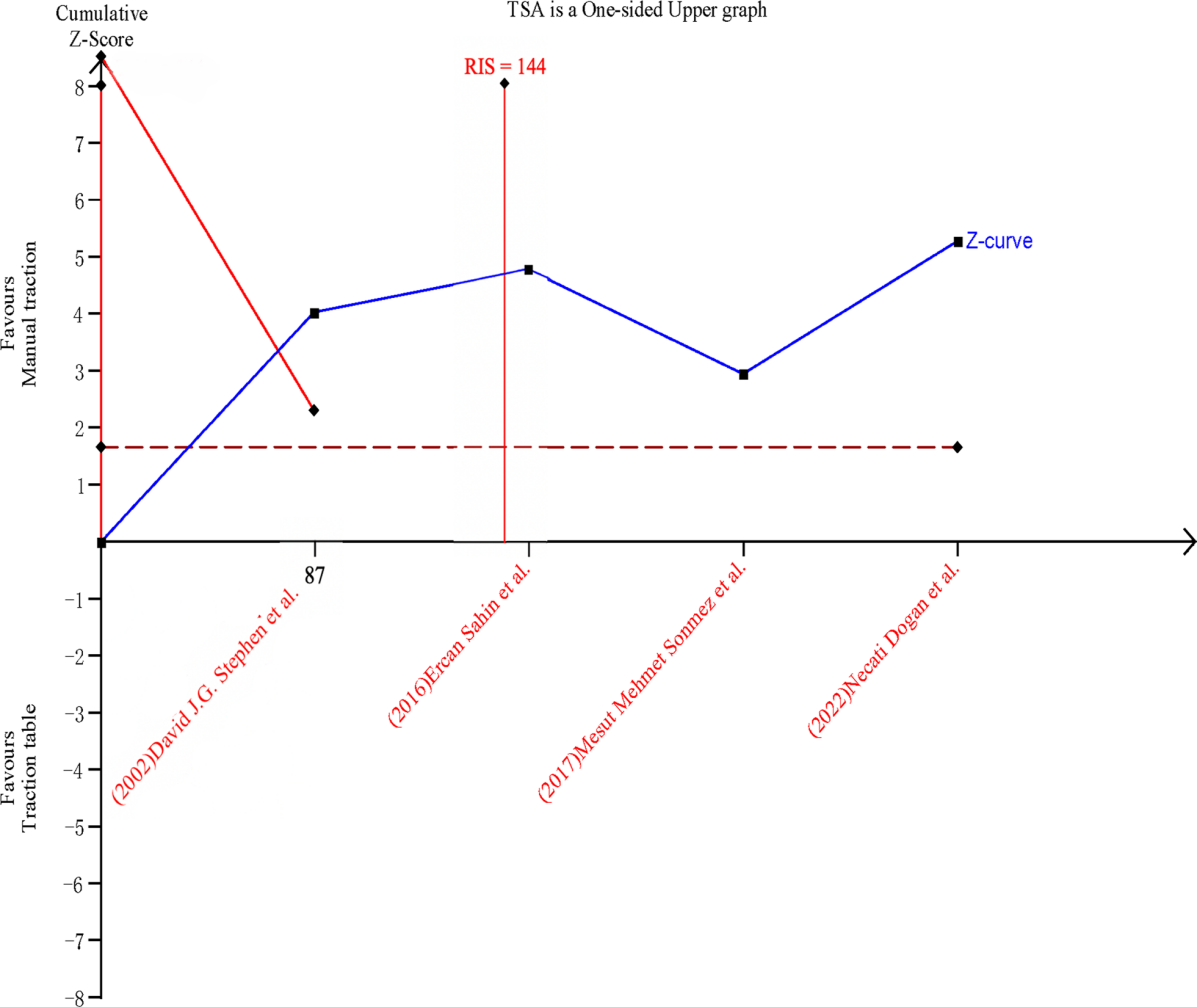
Sequential analysis of the effect on set-up time

The analysis of operative time and set-up time is shown in Fig. 15 and Fig. 16. In the case of α = 0.05 and β = 0.2, the cumulative Z-curve passed the traditional and TSA threshold, verifying the results and avoiding false positive errors. Besides, they all pass through the vertical line of required information size (RIS), indicating that the amount of data can fully prove that manual traction groups have less operative time and set-up time.

## Discussion

The meta-analysis suggested that after a sufficient follow-up period (six months to about 2 years), the traction table showed no significant advantages in fracture healing time, Harris score, and postoperative fracture malunion rate in the femoral fractures patients. Instead, it prolonged the operative time and set-up time. Moreover, it proved that using a traction repositor could reduce fracture healing time.

Manual traction leaves out preoperative traction time, and its routine disinfection and draping are more manageable, which undoubtedly could reduce the set-up time [9]. Manual traction also has advantages in operative time. Even though the intraoperative manual traction was counted, the manual traction group still takes less time than the traction table group, which is not affected by surgery in the supine or lateral position [10]. For lower limb fractures, the traction table easily leads to excessive traction, making unstable fracture fragments shift or turning stable fractures into unstable ones during the insertion of the intramedullary nail. Regarding manual traction, doctors could move the affected limb to coordinate with the intramedullary nail, reducing operation difficulty [11, 12]. It is reasonable that manual traction groups have shorter operative times.

The fracture healing time in the traction repositor group is lower, maybe because it could better reduce the fracture. On the one hand, compared with manual traction, a traction repositor can generate enough force to reduce overlapping deformities. On the other hand, compared with the traction table, the traction repositor can easily be adjusted during surgery to correct rotation deformities [13]. Besides, it is cheaper than the traction table and could be an ideal substitute in community hospitals [14]. Considering that there is no significant difference in prognosis, it is also one of the viable options. However, the number of related studies is insufficient, and this conclusion should be treated cautiously. Using a traction repositor will cause additional damage to the patient, which should also be considered carefully [15].

This study has some limitations: 1. The lack of a high-quality study and the small sample size. Although TSA analysis confirmed the reliability of our results, more high-quality, multi-center, and large-sample RCTs are still needed to verify the conclusions of this study. 2. The included studies’ experimental designs were inconsistent, which would cause a particular risk of bias and eventually affect our conclusions' reliability. 3. Screened studies were limited to English and Chinese, and many took place in China. Hence, the results might be biased in language and ethnicity, requiring more multilingual, multi-regional clinical trials to promote our conclusions.

However, femoral intramedullary nail surgery without a traction table can significantly alleviate patients' discomfort and irritation, relieve their pain and provide a more cost-effective and straightforward surgical plan, which is worthy of further study [16, 17]. The pros and cons should be carefully weighed in clinical, and the most appropriate surgical method should be selected according to every patient's situation.

## Conclusions

The study proved that the traction table has no obvious advantage in improving patient outcomes in the femoral intramedullary nailing surgery. Operation without a traction table can be chosen for simplifying surgery and reducing costs (Additional file 3).

## Supplementary Information

**Additional file 1**.** Table 1**: Detailed search strategy for the PubMed, Embase, Web of Science, and Cochrane Library databases.

**Additional file 2**.** Table 2**: Detailed NOS scores for the individual included cohort studies.

**Additional file 3**. **Appendix Figure 1**: PRISMA Flow Diagram.

## Data Availability

The datasets used and analyzed during the current study are available from the corresponding author on reasonable request.

## Abbreviations

CI: Confidence interval
GRADE: Grade of recommendations assessment, development, and evaluation
NOS: Newcastle–Ottawa scale
PRISMA: Preferred reporting items for systematic reviews and meta-analyses
RCT: Randomized controlled trial
RR: Risk ratio
SMD: Standardized mean difference
TSA: Trial sequential analysis
