# Supplementary material for: Is the traction table necessary to treat femoral fractures with intramedullary nailing? A meta-analysis
Source: J Orthop Surg Res. 2023 Apr 5;18:277. doi: 10.1186/s13018-023-03659-y (PMC10074654; doi:10.1186/s13018-023-03659-y)
Supplement: Supplementary file 1 — Additional file 1. Table 1: Detailed search strategy for the PubMed, Embase, Web of Science, and Cochrane Library databases. [file 13018_2023_3659_MOESM1_ESM.docx]

Appendix Table 1. Detailed search strategy in PubMed, Embase, Web of Science, and Cochrane Library

Source: PubMed; Searched on: August 30, 2022; Results: 661

| **Search number** | **Query** | **Results** |
| --- | --- | --- |
| **1** | femor*[Title/Abstract] OR femur*[Title/Abstract] OR intertrochanter*[Title/Abstract] OR subtrochanter*[Title/Abstract] | 206,283 |
| **2** | fractur*[Title/Abstract] | 298,322 |
| **3** | tract*[Title/Abstract] | 450,050 |
| **4** | bed[Title/Abstract] OR table[Title/Abstract] OR frame[Title/Abstract] | 277,507 |
| **5** | lateral[Title/Abstract] | 334,419 |
| **6** | #3 AND #4 | 5,212 |
| **7** | #5 OR #6 | 339,093 |
| **8** | #1 AND #2 AND #7 | 3,422 |
| **9** | nail*[Title/Abstract] | 37,697 |
| **10** | #8 AND #9 | 661 |

Source: Embase; Searched on: August 30, 2022; Results: 840

| **Search number** | **Query** | **Results** |
| --- | --- | --- |
| **1** | femor*:ab,ti OR femur*:ab,ti OR intertrochanter*:ab,ti OR subtrochanter*:ab,ti | 278431 |
| **2** | fractur*:ab,ti | 361814 |
| **3** | tract*:ab,ti | 596132 |
| **4** | bed:ab,ti OR table:ab,ti OR frame:ab,ti | 735370 |
| **5** | lateral:ab,ti | 418083 |
| **6** | #3 AND #4 | 18537 |
| **7** | #5 OR #6 | 435739 |
| **8** | #1 AND #2 AND #7 | 4568 |
| **9** | nail*:ab,ti | 49146 |
| **10** | #8 AND #9 | 840 |

Source: Web of science; Searched on: August 30, 2022; Results: 1175

| **Search number** | **Query** | **Results** |
| --- | --- | --- |
| 1 | TS=(femor* or femur* or intertrochanter* or subtrochanter*) | 345,473 |
| 2 | TS=(fractur*) | 1,061,591 |
| 3 | TS=(tract*) | 958,143 |
| 4 | TS=(bed or table or frame) | 1,448,309 |
| 5 | TS=(lateral) | 695,527 |
| 6 | #3 AND #4 | 17,689 |
| 7 | #5 OR #6 | 712,098 |
| 8 | #1 AND #2 AND #7 | 5,329 |
| 9 | TS=(nail*) | 90,408 |
| 10 | #8 AND #9 | 1,175 |

Source: Cochrane Library; Searched on: August 30, 2022; Results: 88

| **Search number** | **Query** | **Results** |
| --- | --- | --- |
| 1 | (femor* or femur* or intertrochanter* or subtrochanter*):ti,ab,kw | 21155 |
| 2 | (fractur*):ti,ab,kw | 26371 |
| 3 | (tract*):ti,ab,kw | 46244 |
| 4 | (bed or table or frame):ti,ab,kw | 58334 |
| 5 | (lateral):ti,ab,kw | 18691 |
| 6 | #3 AND #4 | 2359 |
| 7 | #5 OR #6 | 20963 |
| 8 | #1 AND #2 AND #7 | 427 |
| 9 | (nail*):ti,ab,kw | 3797 |
| 10 | #8 AND #9 | 88 |
