# Supplementary material for: Is the traction table necessary to treat femoral fractures with intramedullary nailing? A meta-analysis
Source: J Orthop Surg Res. 2023 Apr 5;18:277. doi: 10.1186/s13018-023-03659-y (PMC10074654; doi:10.1186/s13018-023-03659-y)
Supplement: Supplementary file 2 — Additional file 2. Table 2: Detailed NOS scores for the individual included cohort studies. [file 13018_2023_3659_MOESM2_ESM.docx]

| **Appendix Table 2. Quality assessment using Newcastle-Ottawa Scale** | | | | | | | | | |
| --- | --- | --- | --- | --- | --- | --- | --- | --- | --- |
| Study | Selection | | | | Compar-  -ability | Outcome | | | Score |
|  | Exposed Cohort | Nonexposed Cohort | Ascertainment of Exposure | Interested  Outcome |  | Assessment of Outcome | Length of Follow-up | Adequacy of Follow-up |  |
| Ruipeng Zhang et al. 2018  Du Gangqiang et al. 2020 | *  * | *  * | *  * | *  * | *  * | *  * | *  * | -  - | 7  7 |
| Kuo Zhao et al. 2020 | * | * | * | * | ** | * | * | - | 8 |
| Mingming Yan et al. 2021 | * | * | * | * | * | * | * | - | 7 |
